# Draft Genome Sequence of a Pasteurella multocida Strain Isolated from a Spotted Deer (Axis axis) in India

**DOI:** 10.1128/mra.01297-22

**Published:** 2023-05-11

**Authors:** Akanksha Yadav, Bhoj Raj Singh, Abhijit M. Pawde, Prasad Thomas, Vidya Singh, Rohit Singh, Shiv Varan Singh, Karthikeyan Ravichandran, Himani Agri, Varsha Jayakumar, Raghavendra G. Amachawadi

**Affiliations:** a Department of Epidemiology, ICAR-Indian Veterinary Research Institute, Izatnagar, India; b Centre for Wildlife, ICAR-Indian Veterinary Research Institute, Izatnagar, India; c Department of Bacteriology and Mycology, ICAR-Indian Veterinary Research Institute, Izatnagar, India; d Department of Pathology, ICAR-Indian Veterinary Research Institute, Izatnagar, India; e Department of Clinical Sciences, College of Veterinary Medicine, Kansas State University, Manhattan, Kansas, USA; Loyola University Chicago

## Abstract

Here, we report the genome sequence of a Pasteurella multocida strain isolated from the heart blood of a spotted deer (Bareilly, India). The 2.44-Mbp genome has 2,227 coding sequences, with a G+C content of 40.7%.

## ANNOUNCEMENT

Pasteurella multocida is a Gram-negative bacterium with a broad host range, including poultry, livestock, and wild animals. In livestock, mainly cattle and buffaloes, P. multocida is one of the common bacteria associated with an economically important production disease, bovine respiratory disease complex, and highly lethal hemorrhagic septicemia (1, 2). There are several sporadic reports of P. multocida in wild animals, including spotted deer and leopards (3–6). Although several conventional and molecular typing methods, such as enterobacterial repetitive intergenic consensus (ERIC)/repetitive extragenic palindromic (REP)/randomly amplified polymorphic DNA (RAPD) PCR, Southern blot hybridization, pulse field gel electrophoresis (PFGE), and multilocus sequence typing (MLST) have been largely used in outbreak investigations, very few attempts have been made to sequence the whole genome of a P. multocida strain from wild animals (7, 8). The first complete genome sequence of P. multocida strain Pm70 from an avian host was published in 2001 (9). Currently, there are 382 genome assemblies and annotation reports of P. multocida, primarily of bovine origin, available at NCBI. A heart blood sample from a spotted deer (National Zoological Park, Bareilly, India) was streaked in triplicate onto 5% sheep blood agar plates. The three plates were incubated separately, aerobically at 37°C for 24 h, microaerobically (5% CO_2_) at 37°C for 48 h, and anaerobically in jars with 90% H_2_ plus 10% CO_2_ and palladium catalyst at 37°C for 48 h. Based on the morphology, three to five colonies were selected and confirmed both by biochemical (catalase, oxidase, indole, MR-VP, citrate) and *kmt* gene-specific PCR assays (10). A single pure colony was inoculated into 5 mL of Luria-Bertani broth (HiMedia, Bengaluru, Karnataka) and incubated overnight at 37°C. The overnight bacterial culture was subjected to genomic DNA isolation using the QIAmp DNA minikit (Qiagen, Germantown, MD), as per the manufacturer’s protocol. The purity of the DNA was determined using a NanoDrop spectrophotometer (Thermo Fisher Scientific, Waltham, MA).

Sequencing was performed using the Illumina HiSeq X Ten platform (AgriGenome, Kochi, Kerala, India). Both paired-end and mate-pair libraries with 150-bp reads were generated. Sequence adaptors and low-quality reads were filtered using AdapterRemoval version 2.3.1 (https://github.com/mikkelschubert/adapterremoval). In total, 9,526,396 paired-end and 3,462,016 mate-pair reads were recovered using FastUniq. *De novo* assembly was performed using ABySS version 2.1.5, with the k-mer parameter ranging from 31 to 95 (https://github.com/bcgsc/abyss) (11).

The assembly metrics included the following: genome size, 2,429,285 bp; genome coverage, 40.0×; number of contigs, 50; G+C content, 40.5%; contig *N*_50_, 194,634 bp; contig *L*_50_, 5; number of clustered regularly interspaced short palindromic repeats (CRISPRs), 3. Gene prediction using the NCBI Prokaryotic Genome Annotation Pipeline (PGAP) version 6.3 (12) resulted in a total of 2,316 genes (2,247 coding DNA sequences [CDSs], 10 rRNAs, and 54 tRNAs) and 2,227 functional proteins. Based on the Pathosystems Resource Integration Center (PATRIC) annotation statistics (13), this strain possesses 30 antimicrobial resistance (AMR) determinants (as shown in Table 1) (14) and 8 virulence factors (15). On the basis of the Rural Industries Research and Development Corporation (RIRDC) MLST scheme for P. multocida, the strain belonged to sequence type 122 (ST122). However, the multihost MLST scheme identified a new *deoD* allele (48), and the isolate was assigned to a new ST (ST194). Multilocus sequence typing (MLST) analysis was performed using MLST 2.0 (https://cge.cbs.dtu.dk/services/MLST/) (16). Unless otherwise noted, default parameters were used for all software tools.

**TABLE 1:** Antimicrobial resistance genes with their respective mechanisms determined using a k-mer analysis

AMR mechanism	Gene(s)
Antibiotic inactivation enzyme	*aph(3″)-I*, *aph(6)-Ic*/*aph(6)-Id*
Antibiotic target in susceptible species	*alr*, *ddl*, *dxr*, EF-G, EF-Tu, *folA*, *dfr*, *folP*, *gyrA*, *gyrB*, *inhA*, *fabl*, iso-tRNA, *murA*, *rho*, *rpoB*, *rpoC*, S10p, S12p
Efflux pump conferring antibiotic resistance	*floR* family, *macA*, *macB*, *tet*(A)
Gene conferring resistance via absence	*gidB*
Protein altering cell wall charge conferring antibiotic resistance	*gdpD*, *pgsA*
Regulator modulating expression of antibiotic resistance genes	H-NS, *oxyR*

### Data availability.

This whole-genome shotgun sequencing project has been submitted to NCBI and is available at GenBank under the accession number JACGZI000000000. The version described in this paper is version JACGZI000000000.1. The Illumina reads are available in the Sequence Read Archive (SRA) under the accession number SRR12182089.
